# Assessing general hospital doctors’ attitudes toward psychiatric care in multicultural settings

**DOI:** 10.1186/s13104-024-06788-7

**Published:** 2024-05-02

**Authors:** Inoka Wimalaratne, Jessica McLay, David B Menkes

**Affiliations:** 1 Department of Psychological Medicine, University of Auckland, Auckland, New Zealand; 2 Monash Health Mental Health Programme, 126-128, Cleeland Street, Dandenong Vic, Melbourne, 3175 Australia; 3 Department of Statistics, University of Auckland, Auckland, New Zealand

**Keywords:** Hospital doctors, Attitudes, Psychiatric care, Mental illness, Culture

## Abstract

**Objective:**

Psychiatric care in general hospitals depends on collaboration with non-psychiatrist doctors. The Doctors’ Attitudes toward Collaborative Care for Mental Health (DACC-MH) is a two-factor scale designed to address this issue and validated in the UK in 2010. However, its applicability in contemporary, culturally diverse settings is unknown and therefore this study was aimed at determining its validity and consistency using data from our 2021 international study. Confirmatory and exploratory factor analyses were used, comparing results from our 2021 study (*n* = 889) with those from the 2010 UK study (*n* = 225).

**Results:**

The DACC-MH consultation subscale, but not the management subscale, aligned with data from our larger, international study. The 2-factor model failed the Chi-square goodness of fit test (χ^2^(19) = 53.9, *p* < 0.001) but had acceptable other fit indices. While the previously identified attitudinal difference between physicians and surgeons was replicated, measurement invariance for this result could not be established. Exploratory factor analysis suggested a 6-factor model, contrasting with the 2-factor model proposed in 2010 for the UK sample. The DACC-MH scale shows significant limitations when applied to a larger, international dataset. Cultural and generational differences in doctors’ attitudes appear relevant and should be considered in assessing barriers to psychiatric care in general hospitals.

**Supplementary Information:**

The online version contains supplementary material available at 10.1186/s13104-024-06788-7.

## Introduction

Psychiatric comorbidity in medically ill populations is associated with increased mortality and morbidity, functional impairment, loss of productivity, longer hospital stays, and increased use of health services [1–4]. These findings highlight the potential importance of psychiatric care in general hospital settings. Consultation-liaison psychiatry (CLP) is a recognised model for providing timely and evidence based psychiatric care in general hospitals, but funding and management remain a challenge. Studies have shown that provision of CLP services varies in different hospital settings [5–7] and depends on the differing views of frontline clinicians who are the main source of referrals [8]. Attitudes of general hospital doctors toward CLP and other collaborative care models [9] are thus pivotal to their implementation and outcomes.

Collaborative care in this case includes two key components: mental health management by non-psychiatric health professionals and their consultation with psychiatrists [9]. Attitudes of hospital doctors toward mental health management and their willingness to consult with psychiatrists have been reported to vary by specialty, seniority, gender and cultural setting [10–15]. In 2010, Thombs and colleagues developed the 8-item Doctors’ Attitudes Toward Collaborative Care for Mental Health (DACC-MH) scale, with two hypothesised factors, management and consultation, validated by confirmatory factor analysis (CFA) [12]. Data were taken from a previous study [11] at a teaching hospital in London in 2003 (*n* = 225), using an original questionnaire (41 items) developed by Mayou and Smith in 1986 [10]. Thombs’ analysis tested known-groups validity and found physicians scored higher than surgeons on all 8 items, indicating more favourable attitudes toward both consultation and management.

The influence of culture on medical attitudes is illustrated by recent studies of doctors practising in various countries [13–15]. Since the DACC-MH scale was developed only in the UK, the aim of this study was to determine its applicability and psychometric properties in other settings.

## Methods

We used data from our study [15] of 889 hospital specialists based in seven culturally distinct countries (China, New Zealand, Sri Lanka, Israel, Brazil, Russia, and the Netherlands) based on the original questionnaire [10] from which the DACC-MH was derived. The total sample of responders was characterised by equal gender distribution and a majority (51%) working as physicians/ internal medicine specialists. Statistical analyses were performed using the statistical package R [16].

In a first step, we conducted confirmatory factor analysis (CFA) of the previously reported 2-factor model to compare results from our international data with those from the 2003 UK dataset. Secondly, we used exploratory factor analysis (EFA) to determine the optimal factor structure for our international sample.

Each of the items used in both the CFA and EFA allowed respondents a binary choice with which they could either agree or disagree. Fifty-eight (6.5%) of the participants were missing at least one of the eight CFA items so a multiple imputation approach was used (see Supplementary File 1). The CFA was conducted using procedures for ordinal (including dichotomous) data.

We examined Cronbach’s alphas, a measure of internal consistency, for the consultation and management scales and compared these with Thombs’ report.

We also examined the measurement invariance of the CFA (see Supplementary File 1) and ran the 2-factor model on each country individually using the original dataset with listwise deletion.

Testing for measurement invariance of Thombs’ 2-factor model comparing physicians and surgeons was not possible because the model converged for physicians but not surgeons. We fit a model with just the consultation factor to the surgeon data and provide the results for this along with the results for the 2-factor model for physicians.

Similar to Thombs et al., we calculated consultation factor sum scores (maximum 4) for each participant, using a one-way analysis of variance to compare physicians, surgeons, and other doctors. Comparable analysis of management scale data was impossible due to low internal consistency and lack of between groups measurement invariance.

For the EFA, seven questions attracting more than 90% participant agreement were excluded, consistent with Thombs’ method, ensuring included items had sufficient variance to usefully differentiate participant attitudes. It also helped make the data more appropriate for factor analysis increasing the Kaiser-Meyer Olkin (KMO) from 0.15 to 0.63. An exception was made for the question ‘I would like to know more about what psychiatrists have to offer in the management of medical or surgical patients’. This had a percentage agreement of 90.2% but was included in the EFA because it was one of the final items in Thombs’ CFA.

To determine the suitability of the dataset for factor analysis, KMO values were examined and Bartlett’s test of sphericity performed. Examination of the scree plot and parallel analysis (using the fa.parallel function (psych package)) led us to choose 6 factors. EFAs were also done separately for each country (see Supplementary File 1).

## Results

### Part 1: confirmatory factor analysis (CFA)

The original Cronbach alphas for Thombs’ data were marginal at 0.67 and 0.65 for the consultation and management factors, respectively. For our data, the Cronbach alpha was comparable (0.65) for consultation but unacceptable (0.34) for management.

The indices in Table 1 indicate adequate fit of the 2-factor model to the international data; although examination of factor loadings (Table 2) shows relatively modest loadings for Management Scale items, raising concern about the applicability of this model to the data. The loadings for the Consultation Scale were better, all about 0.7 or higher, more consistent with the UK data.

**Table 1:** Goodness of Fit measures for the CFA

Measure/Statistic	Rule of thumb for good fit	UK study: full model	International study: full model	International study: model excluding Management
Chi-Square for goodness of fit	*p* > 0.05	χ^2^(11) = 12.7, *p* = 0.31	χ^2^(19) = 53.9, *p* < 0.001	χ^2^(2) = 9.8, *p* = 0.007
Comparative fit index (CFI)	> 0.9	0.99	0.935	0.982
Tucker-Lewis index (TLI)	> 0.9	0.99	0.904	0.945
Root mean square of approximation (RMSEA) (90% CI)	< 0.05	0.03	0.045 (0.031, 0.06)	0.066 (0.029, 0.11)

**Table 2:** Comparison of standardised item factor loadings and percentage of participants agreeing with each item

Item number	Item Wording	Factor loadings (10 imputed datasets)		Percentage agreeing (non-imputed dataset after excluding missing data)	
		UK study	International study	UK study	International study
*Consultation scale*					
Q3	I would welcome more contact with psychiatrists	0.59	0.74	68%	85%
Q33	I would like more help in providing psychological and social care	0.67	0.69	76%	87%
Q35	I would like to know more about what psychiatrists have to offer in the management of medical or surgical patients	0.82	0.71	80%	90%
Q39	I would like more contact with the psychiatric service	0.92	0.89	79%	85%
*Management scale*					
Q11	Management of emotional problems is an important part of my care of chronic outpatients	0.62	0.68	74%	82%
Q31 (reversed)	When psychological factors appear to be an important cause of the presenting problem, I confine myself to physical assessment	0.97	0.36	84%	81%
Q34	Hospital doctors should be able to use psychological methods like discussion of anxiety/problems	0.91	0.51	91%	88%
Q37 (reversed)	Hospital doctors are not responsible for emotional care of patients	0.47	0.26	84%	83%

The CFA was also completed using only the Consultation Scale to determine if a better fit resulted. The factor loadings for these items remained roughly the same and the fit statistics improved overall (see Table 1).

The 2-factor CFA model was only able to converge for New Zealand, Brazil, Russia, and the Netherlands, hence the testing of measurement invariance only proceeded with these countries (see supplementary File 1). The four-country model with no equality constraints (to test configural invariance) was found to be on the borderline of acceptable fit (χ^2^(76) = 121.1, *p* = 0.001, CFI = 0.93, TLI = 0.90, RMSEA = 0.07, 90% CI=(0.04, 0.09)).

The single-factor consultation model for surgeons fit well (χ^2^ (2) = 3.40, *p* = 0.183, CFI = 0.99, TLI = 0.98, RMSEA = 0.07, 90% CI=(0.00, 0.19)) as did the 2-factor model for physicians (χ^2^(19) = 23.01, *p* = 0.237, CFI = 0.96, TLI = 0.93, RMSEA = 0.03, 90% CI=(0.00, 0.06)). Given these results we would not assume metric (and hence scalar) invariance between physicians and surgeons.

The mean consultation score was 3.3 for surgeons and 3.7 for physicians (*p* < 0.0001). Although we did not establish measurement invariance for the consultation scale, we did perform the comparison in sum scores between physicians and surgeons in order to provide a comparison to Thombs’ analysis (see Supplementary File 1).

### Part 2: exploratory factor analysis (EFA)

The dataset (after removing items with greater than 90% agreement) was found to be suitable for exploratory factor analysis. The overall KMO index was 0.63 and most items (18 out of 22) had individual KMO values greater than 0.5. Bartlett’s test of sphericity was significant (χ^2^ (231) = 833, *p* < 0.0001) providing strong evidence that the matrix of correlations was not a matrix of zeros.

**Table 3:** Factor loadings

Factor 1		Factor 2		Factor 3		Factor 4		Factor 5		Factor 6	
12% variance extracted		10% variance extracted		9% variance extracted		7% variance extracted		6% variance extracted		6% variance extracted	
Q	Loading	Q	Loading	Q	Loading	Q	Loading	Q	Loading	Q	Loading
3	0.77	2	0.31	34	0.80	36	0.98	4	0.54	12	0.92
28	0.39	8	0.59	37	-0.39			5	0.90	31	-0.31
35	0.62	9	0.49	38	0.57						
39	0.67	11	0.83	41	0.43						
33	0.84	13	-0.39								

The 6 factors from the EFA explained 50% of the variance in the data. This 6-factor model was compared with Thombs’ 2-factor model (Table 3). Factor 1 from our data was almost the same as Thombs’ consultation factor with questions 3, 33, 35, and 39 loading onto the factor; question 28 (“I would welcome more time to talk to my patients”) rather less so. Factor 3 had some similarity to Thombs’ management factor, with questions 34 and 37 loading onto the factor, but not 11 or 31 as found in his study. The questions loading onto Factors 2 and 4–6 were examined and theoretically aligned more with attitudes towards management than consultation.

The number of factors in the individual country CFAs varied from 4 to 8 (see Supplementary File 1). Thombs’ Consultation Factor was reproduced in the Russian data with the same four questions (3, 33, 35 and 39) falling onto Factor 1, with similar results seen for Brazil, Israel, and The Netherlands. By contrast, the questions from Thombs’ Management Factor appeared with various other factors, spread across our seven countries’ EFAs.

## Discussion

In testing the validity of the DACC-MH scale using CFA, our international dataset supported the consultation but not the management subscale. This finding indicates that the 2-factor model developed by Thombs et al. does not adequately describe our large, culturally diverse sample.

We used EFA to examine the factor structure in our international data, both overall and in individual country samples. In contrast to Thombs’ model, there was evidence for more than two factors in the total sample, with only the first factor aligning to Thombs consultation factor. Analysis of individual country samples also showed little consistency with Thombs’ 2-factor model and suggested the presence of other factors, however, given the small sample sizes for individual countries, it was not possible to accurately estimate these factor structures. In addition, our results are subject to the limitations inherent to an EFA. There are many methods available for factoring and rotating; each comes with advantages and disadvantages and may generate different results. There are also different ways to choose the number of factors. Hence the results from our EFA should not be considered the only plausible outcome. Since random data can produce factors in an EFA, it can be difficult to assess how much a factor reflects real-world patterns versus random variation in the data.

The DACC-MH scale was based on a questionnaire developed in 1986 [10] and used in a UK survey conducted in 2003 [11]. Although ethnic data were not reported, it is highly likely this previous sample was less culturally diverse than our international dataset [15]. In addition to culture, there may be other variables influencing the limited applicability of this scale to our data, such as changes in clinical practice, service delivery models, and medical training over the intervening years.

Previously identified differences between physicians and surgeons using sum scores [12] was replicated in our analysis. As these differences have also been observed in other studies [10–15], our findings strengthen evidence for an enduring attitudinal difference between these two medical disciplines. However, we were not able to demonstrate measurement invariance between physicians and surgeons so we could not establish that the use of Thombs’ 2-factor model was a meaningful way to compare these groups. Additionally, our assessable surgeon group included in the model was small (*n* = 151), drawn from multiple countries for which measurement invariance had not been demonstrated, complicating both assessment of invariance and comparison of physicians and surgeons.

The finding that the CFA was a better fit on Thombs’ data compared to the international data is unsurprising, given that the UK data was sample from which the model was derived. Thombs’ started with 15 items (5 consultation and 10 management) in the CFA and followed a process (removing items with greater than 90% agreement or with factor loadings less than 0.4), resulting in the published 2-factor model [12]. The fact that the loadings for the management subscale were significantly lower suggests that this factor may be specific to his data and not generalizable to other countries and time periods.

## Conclusion

The DACC-MH scale developed in the UK has limited applicability and consistency when applied to our larger, international dataset. Cultural differences in attitudes as well as changes in service delivery models over the last decade are possible explanations. Further studies are needed to develop a better understanding of attitudes of general hospital doctors in culturally diverse settings towards management of psychiatric comorbidities. Such understanding would help to identify barriers and solutions to the provision of psychiatric care in general hospitals internationally. For example, it could enable delivery of culturally appropriate and tailored education for non-psychiatric medical professionals in order to improve practice and promote optimal psychiatric care in the general hospital setting.

### Electronic supplementary material

Below is the link to the electronic supplementary material.

Supplementary Material 1

## Data Availability

The datasets used and analysed during the current study are available from the corresponding author on reasonable request.

## Abbreviations

DACC: MH-Doctors’ attitudes toward collaborative care for mental health
CLP: Consultation-liaison psychiatry
CFA: Confirmatory factor analysis
EFA: Exploratory factor analysis
CLI: Comparative fit index
TLI: Tucker-Lewis index
RMSEA: Root mean square of approximation
