# Supplementary material for: Assessing general hospital doctors’ attitudes toward psychiatric care in multicultural settings
Source: BMC Res Notes. 2024 May 2;17:125. doi: 10.1186/s13104-024-06788-7 (PMC11067133; doi:10.1186/s13104-024-06788-7)
Supplement: Supplementary file 1 — Supplementary Material 1 [file 13104_2024_6788_MOESM1_ESM.docx]

**Supplementary files**

*Table 1: Percentage agreeing with target statements out of those that answered the question*

| Question | 1 | 2 | 3 | 4 | 5 | 6 | 7 | 8 | 9 | 10 | 11 | 12 | 13 | 14 | 15 |
| --- | --- | --- | --- | --- | --- | --- | --- | --- | --- | --- | --- | --- | --- | --- | --- |
| N answered | 888 | 889 | 889 | 886 | 888 | 885 | 880 | 883 | 882 | 885 | 878 | 880 | 882 | 875 | 879 |
| N missing | 1 | 2 | 0 | 3 | 1 | 4 | 9 | 6 | 7 | 4 | 11 | 9 | 7 | 14 | 10 |
| Percent of non-missing agreeing | 96% | 33% | 85% | 42% | 22% | 94% | 62% | 59% | 68% | 99% | 82% | 11% | 53% | 60% | 92% |

| Question | 28 | 29 | 30 | 31 | 32 | 33 | 34 | 35 | 36 | 37 | 38 | 39 | 40 | 41 |
| --- | --- | --- | --- | --- | --- | --- | --- | --- | --- | --- | --- | --- | --- | --- |
| N answered | 843 | 841 | 837 | 841 | 843 | 842 | 840 | 839 | 837 | 840 | 836 | 839 | 839 | 837 |
| N missing | 46 | 48 | 52 | 48 | 46 | 47 | 49 | 50 | 52 | 49 | 53 | 50 | 50 | 52 |
| Percent of non-missing agreeing | 88% | 90% | 79% | 19% | 95% | 87% | 88% | 90% | 86% | 17% | 56% | 85% | 10% | 81% |

**Missing data**

Ten imputed datasets were created with the R function mice using logistic regression. Using the runMI function (semTools package), the CFA was run separately for each imputed dataset (using the cfa function (lavaan package) and the results pooled using Rubin’s rules. ^16^ The results from the multiple imputation approach were compared to those from using listwise exclusion (a participant was excluded if they had a missing value for any of the eight items used in Thomb’s 2-factor model).

*Table 2: Comparison of model fit statistics from different missing value approaches*

| Measure/Statistic | Multiple Imputation | Listwise exclusion |
| --- | --- | --- |
| Chi-Square for goodness of fit | χ^2^(19)=53.9, p<0.001 | χ^2^(19)=61.6,  p<0.001 |
| Comparative fit index (CFI) | 0.935 | 0.947 |
| Tucker-Lewis index (TLI) | 0.904 | 0.922 |
| Root mean square of approximation (RMSEA)  (95% CI) | 0.045  (0.031, 0.060) | 0.052  (0.038, 0.067) |

*Table 3: Comparison of standardised item factor loadings from different missing value approaches*

| Item number | Item Wording | Standardised Factor Loading | |
| --- | --- | --- | --- |
|  |  | Multiple Imputation | Listwise exclusion |
| *Consultation scale* | |  |  |
| Q3 | I would welcome more contact with psychiatrists | 0.74 | 0.73 |
| Q33 | I would like more help in providing psychological and social care | 0.69 | 0.69 |
| Q35 | I would like to know more about what psychiatrists have to offer in the management of medical or surgical patients | 0.71 | 0.73 |
| Q39 | I would like more contact with the psychiatric service | 0.89 | 0.89 |
| *Management scale* | |  |  |
| Q11 | Management of emotional problems is an important part of my care of chronic outpatients | 0.68 | 0.66 |
| Q31 (reversed) | When psychological factors appear to be an important cause of the presenting problem, I confine myself to physical assessment | 0.36 | 0.34 |
| Q34 | Hospital doctors should be able to use psychological methods like discussion of anxiety/problems | 0.51 | 0.51 |
| Q37 (reversed) | Hospital doctors are not responsible for emotional care of patients | 0.26 | 0.25 |

**Measurement invariance**

We examined the measurement invariance of the CFA and ran the 2-factor models on each country individually using the original dataset with listwise deletion. This was justified due to the multiple imputation results being very similar to those from excluding missing data in a list-wise fashion.

Configural invariance was tested for the 2-factor CFA model by fitting a multi-group CFA where the model was fitted separately for each country without any equality constraints. This provided a test of whether the same factorial structure held across all countries. The multi-country CFA could not be fit with all seven countries as the model did not converge for China, Sri Lanka, and Israel, and so the model was run with just the four remaining countries (New Zealand, Brazil, Russia, and the Netherlands).

Metric invariance (same factor loadings across countries) was tested by constraining the factor loadings to be equal across the four countries (thresholds, equivalent to intercepts for models with continuous variables, were allowed to vary) and then comparing this multi-group model with the unconstrained multi-group model with a scaled Chi-squared difference test.

The scalar model with both the factor loadings and thresholds constrained to be equal across the countries could not be estimated and so the multi group metric and scalar model were run treating the binary variables as continuous. This allowed the models to converge and be tested against each other (again using a scaled Chi-squared difference test) to give some idea of whether scalar invariance was present.

The four-country metric model was a significantly worse fit than the configural model (χ^2^ (18) =30.1, p=0.037) providing no evidence of metric invariance across the four countries. An examination of the factor loadings for the four countries showed that the factor loadings for Russia were notably different to the other three. Therefore, configural and metric multi-country models were re-run with just the three countries. The fit for the three-country configural model was good (χ^2^ (57) =60.6, p=0.346, CFI=0.99, TLI=0.99, RMSEA=0.02, 90% CI=(0.00, 0.06)) and metric invariance also held (χ^2^(12)=16.9, p=0.152). A three-country scalar model (equal factor loadings and intercepts) was found to be a significantly worse fit compared to the corresponding metric model (χ^2^ (12)=74.2, p=<0.0001) providing no evidence for scalar invariance.

*Table 4: Fit statistics for Thombs 2-factor model for the four countries where the model converged.*

| Measure/ Statistic | UK data | International study - All countries | Russia | Brazil | New Zealand | The Netherlands |
| --- | --- | --- | --- | --- | --- | --- |
| Chi-Square goodness of fit test | χ^2^(11)=12.7, p=0.31 | χ^2^(19)=61.6, p<0.001 | χ^2^(19)=60.5, p<0.001 | χ^2^(19)=10.9, p=0.929 | χ^2^(11=9)=30.5, p=0.046 | χ^2^(19)=19.3, p=0.436 |
| CFI | 0.99 | 0.95 | 0.68 | 1.00 | 0.94 | 1.00 |
| TLI | 0.99 | 0.92 | 0.53 | 1.07 | 0.91 | 1.00 |
| RMSEA  (90% CI^*^) | 0.03 | 0.05  (0.03,0 .07) | 0.15  (0.11,0.19) | 0.00  (0.00,0.03) | 0.07  (0.01,0.11) | 0.01  (0.00, 0.07) |

^*^ Confidence interval for the RMSEA was not provided by Thombs for the UK data

*Table 5: Standardised factor loadings from Thomb’s two-factor model for the four countries where the model converged.*

| Item number | Item Wording | UK data | International study - All countries | Russia | Brazil | New Zealand | The Netherlands |
| --- | --- | --- | --- | --- | --- | --- | --- |
| *Consultation scale* | |  |  |  |  |  |  |
| Q3 | I would welcome more contact with psychiatrists | 0.59 | 0.73 | 0.40 | 0.82 | 0.79 | 0.81 |
| Q33 | I would like more help in providing psychological and social care | 0.67 | 0.69 | 0.74 | 0.66 | 0.67 | 0.51 |
| Q35 | I would like to know more about what psychiatrists have to offer in the management of medical or surgical patients | 0.82 | 0.73 | 0.70 | 0.88 | 0.51 | 0.71 |
| Q39 | I would like more contact with the psychiatric service | 0.92 | 0.89 | 0.45 | 0.91 | 0.98 | 0.83 |
| *Management scale* | |  |  |  |  |  |  |
| Q11 | Management of emotional problems is an important part of my care of chronic outpatients | 0.62 | 0.66 | 0.61 | 0.63 | 0.62 | 0.40 |
| Q31 (disagree) | When psychological factors appear to be an important cause of the presenting problem, I confine myself to physical assessment | 0.97 | 0.34 | 0.66 | 0.69 | 0.76 | 0.09 |
| Q34 | Hospital doctors should be able to use psychological methods like discussion of anxiety/problems | 0.91 | 0.51 | 0.27 | 0.65 | 0.78 | 0.63 |
| Q37 (disagree) | Hospital doctors are not responsible for emotional care of patients | 0.47 | 0.25 | 0.22 | 0.72 | 0.74 | 0.10 |

**Comparison between physicians and surgeons**

*Table 6: Comparison of sum scores between physicians and surgeons*

|  |  | Consultation | |  | |
| --- | --- | --- | --- | --- | --- |
|  |  | UK study | International study |  |  |
| Surgeons | mean | 3.1 (SD 1.2) | 3.3 (SD 1.1) |  |  |
|  |  |  |  |  |  |
| Physicians | mean | 3.5 (SD 0.9) | 3.7 (SD 0.6) |  |  |
|  |  |  |  |  |  |
| p-value |  | <0.01 | <0.0001 |  |  |

*Table 7: Standardised factor loadings from the consultation only model for the surgeon data and the two-factor model for the physician data.*

|  | Surgeons  (n=151) | Physicians  (n=341) |
| --- | --- | --- |
| *Consultation Factor:* |  |  |
| Q3 | 0.70 | 0.62 |
| Q33 | 0.76 | 0.49 |
| Q35 | 0.74 | 0.50 |
| Q39 | 0.94 | 0.76 |
| *Management Factor:* |  |  |
| Q11 |  | 0.33 |
| Q31 (reversed) |  | 0.53 |
| Q34 |  | 0.69 |
| Q37 (reversed) |  | 0.57 |

**EFA**

To determine the suitability of the dataset for factor analysis, the KMO values (overall and for each individual item) were examined and Bartlett’s test of sphericity for performed. Examination of the scree plot and parallel analysis results (using the fa.parallel R function in the psych package) led us to choose 6 factors (see Supplementary File). The EFA was performed with the fa function (psych package) with maximum likelihood extraction and oblimin rotation. The EFA, parallel analysis, and scree plot were performed on the matrix of tetrachoric correlations for dichotomous data. EFAs were done separately for each country using the same methodology as for the total sample.

*Figure 1: Parallel analysis scree plot comparing the scree of the factors of the observed data*


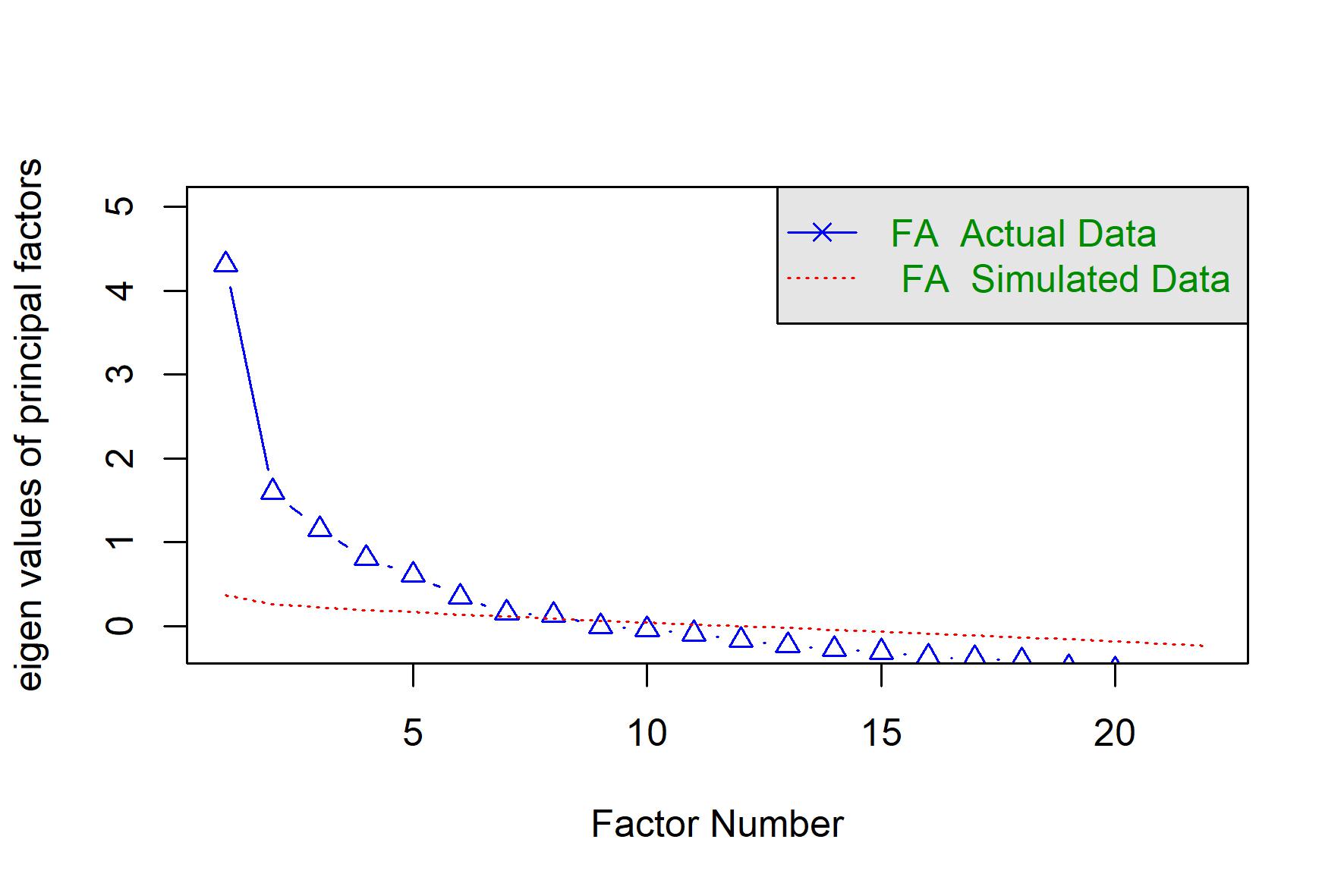


The eigenvalues plotted are those from a principal axis factor analysis using the minimum residual factoring method. Eight eigenvalues were higher than their corresponding random eigenvalues, however the 7^th^ and 8^th^ eigenvalues were very close to the simulated line leading us to choose a six factor model. Number of observations=889.

*Table 8: Factor loading and variance for all seven countries combined (based on the tetrachoric correlation matrix from n=889 participants). The questions listed in the second column are ordered from highest to lowest loading.*

| Factor | Questions loading onto factor | Sum of squared (SS) loadings | Proportion of variance the factor explains | Cumulative variance explained |
| --- | --- | --- | --- | --- |
| 1 | 33, 3, 39, 35, 28 | 2.75 | 0.12 | 0.12 |
| 2 | 11, 8, 9, 13, 2 | 2.13 | 0.10 | 0.22 |
| 3 | 34, 38, 41, 37 | 1.99 | 0.09 | 0.31 |
| 4 | 36 | 1.53 | 0.07 | 0.38 |
| 5 | 5, 4 | 1.33 | 0.06 | 0.44 |
| 6 | 12, 31 | 1.33 | 0.06 | 0.50 |

*Table 9: Summary of EFA results by country*

| Country | Number of Factors | Percent Variance Explained |
| --- | --- | --- |
| New Zealand | 4 | 50% |
| Sri Lanka | 6 | 69% |
| China | 4 | 56% |
| Russia | 5 | 47% |
| Brazil | 6 | 67% |
| Israel | 7 | 75% |
| Netherlands | 8 | 62% |

*Table 10: Factor loading and variance for New Zealand (based on the tetrachoric correlation matrix from n=159 participants). The questions listed in the second column are ordered from highest to lowest loading.*

| Factor | Questions Loading onto Factor | SS Loading | Proportion of Variance the Factor Explains | Cumulative Variance Explained |
| --- | --- | --- | --- | --- |
| 1 | 41, 37, 9, 11, 31, 13, 8, 5, 7, 2 | 3.97 | 0.19 | 0.19 |
| 2 | 33, 3, 36, 30 | 2.54 | 0.12 | 0.31 |
| 3 | 35, 34, 39 | 2.49 | 0.12 | 0.43 |
| 4 | 28, 14 | 1.52 | 0.07 | 0.50 |

*Table 11: Factor loading and variance for Sri Lanka (based on the tetrachoric correlation matrix from n=100 participants). The questions listed in the second column are ordered from highest to lowest loading.*

| Factor | Questions Loading onto Factor | SS Loading | Proportion of Variance the Factor Explains | Cumulative Variance Explained |
| --- | --- | --- | --- | --- |
| 1 | 37, 38, 2 | 2.65 | 0.13 | 0.13 |
| 2 | 34, 29, 31 | 2.57 | 0.12 | 0.25 |
| 3 | 12, 5, 39 | 2.55 | 0.12 | 0.37 |
| 4 | 3, 4, 36, 30 | 2.36 | 0.11 | 0.48 |
| 5 | 33 | 2.31 | 0.11 | 0.59 |
| 6 | 13, 11, 35 | 2.01 | 0.10 | 0.69 |

*Table 12: Factor loading and variance for China(based on the tetrachoric correlation matrix from n=110 participants). The questions listed in the second column are ordered from highest to lowest loading.*

| Factor | Questions Loading onto Factor | SS Loading | Proportion of Variance the Factor Explains | Cumulative Variance Explained |
| --- | --- | --- | --- | --- |
| 1 | 38, 30, 11, 14, 9, 37, 6, 13, 2 | 3.67 | 0.17 | 0.17 |
| 2 | 34, 35, 12, 40 | 3.11 | 0.14 | 0.31 |
| 3 | 5, 4, 39, 29 | 2.75 | 0.13 | 0.43 |
| 4 | 33, 8, 3, 7 | 2.72 | 0.12 | 0.56 |

*Table 13: Factor loading and variance for Russia (based on the tetrachoric correlation matrix from n=100 participants). The questions listed in the second column are ordered from highest to lowest loading.*

| Factor | Questions Loading onto Factor | SS Loading | Proportion of Variance the Factor Explains | Cumulative Variance Explained |
| --- | --- | --- | --- | --- |
| 1 | 39, 35, 3, 33 | 2.71 | 0.12 | 0.12 |
| 2 | 11, 8, 9, 31, 13, 2 | 2.29 | 0.10 | 0.23 |
| 3 | 34, 38, 41, 37, 28 | 2.01 | 0.09 | 0.32 |
| 4 | 5, 12, 4 | 1.77 | 0.08 | 0.40 |
| 5 | 36 | 1.53 | 0.07 | 0.47 |

*Table 14: Factor loading and variance for Brazil (based on the tetrachoric correlation matrix from n=106 participants). The questions listed in the second column are ordered from highest to lowest loading.*

| Factor | Questions Loading onto Factor | SS Loading | Proportion of Variance the Factor Explains | Cumulative Variance Explained |
| --- | --- | --- | --- | --- |
| 1 | 3, 39, 35, 33, 28 | 2.84 | 0.14 | 0.14 |
| 2 | 11, 37, 9, 2, 8 | 2.59 | 0.13 | 0.27 |
| 3 | 36, 31, 13 | 2.55 | 0.13 | 0.40 |
| 4 | 5, 4 | 1.95 | 0.10 | 0.50 |
| 5 | 7, 34, 32 | 1.78 | 0.09 | 0.59 |
| 6 | 38 | 1.63 | 0.08 | 0.67 |

*Table 15: Factor loading and variance for Israel (based on the tetrachoric correlation matrix from n=135 participants). The questions listed in the second column are ordered from highest to lowest loading.*

| Factor | Questions Loading onto Factor | SS Loading | Proportion of Variance the Factor Explains | Cumulative Variance Explained |
| --- | --- | --- | --- | --- |
| 1 | 33, 35 | 2.35 | 0.12 | 0.12 |
| 2 | 34, 39, 36, 14 | 2.25 | 0.11 | 0.23 |
| 3 | 29, 4, 11 | 2.24 | 0.11 | 0.34 |
| 4 | 5, 12 | 2.21 | 0.11 | 0.45 |
| 5 | 7, 2, 37 | 2.19 | 0.11 | 0.56 |
| 6 | 31, 8, 9 | 1.97 | 0.10 | 0.66 |
| 7 | 3, 13 | 1.81 | 0.09 | 0.75 |

*Table 16: Factor loading and variance for the Netherlands (based on the tetrachoric correlation matrix from n=179 participants). The questions listed in the second column are ordered from highest to lowest loading.*

| Factor | Questions Loading onto Factor | SS Loading | Proportion of Variance the Factor Explains | Cumulative Variance Explained |
| --- | --- | --- | --- | --- |
| 1 | 39, 3, 35 | 2.27 | 0.09 | 0.09 |
| 2 | 8, 11, 9, 4, 14 | 1.94 | 0.08 | 0.18 |
| 3 | 29, 7, 2 | 1.90 | 0.08 | 0.25 |
| 4 | 15, 31, 33, 38, 13 | 1.85 | 0.08 | 0.33 |
| 5 | 37, 30, 14 | 1.80 | 0.07 | 0.41 |
| 6 | 28, 41 | 1.75 | 0.07 | 0.48 |
| 7 | 5, 12 | 1.70 | 0.07 | 0.55 |
| 8 | 36 | 1.68 | 0.07 | 0.62 |
